# Dr. Baillie on the Dedication of Dr. Roberon's Book

**Published:** 1816-06

**Authors:** M. Baillie

**Affiliations:** Lower Grosvenor-street


*To the Editors of the London Medical and Physical Journal.*

GENTLEMEN,

A DEDICATION to me being prefixed to Dr. Roberton's Book, I feel it to be necessary, in justice to myself, to give the following public explanation of the circumstances.

About six years ago, Dr. Roberton wrote to me from Scotland, requesting that I would permit him to dedicate a book to me, which he was about to publish. In this book he was to examine some opinions of Sir Everard Home. I consented to accept of the dedication, provided these opinions were examined with liberality, more especially as Dr. Roberton was a native of that parish in which my father had been long established as the clergyman, and where I had spent most of my infant years. When this book was published, I looked into various parts of it, without reading it regularly, and I do not recollect to have observed in it indecency of language. A few days ago, a friend of mine shewed me the third edition of Dr. Roberton's book, in which were a good many passages of the most indecent nature. This struck me with astonishment and indignation. I shall not express what I think of Dr. Roberton's conduct in prefixing my name to an obscene book, without my knowledge; but, from the obscenity itself, he has forfeited his character and rank in society. I am anxious, however, to preserve mine, and therefore think it necessary to communicate, through your Journal, that I did not know there was either a second or a third edition of this book, till my friend shewed to me the third edition; and that I even thought Dr. Roberton had been dead for the last two years. I have now mentioned every thing which I know relative to this most disagreeable business; and I trust that from the tenour of my public and private life, I shall not be readily suspected of encouraging so gross a violation of morality and decorum.

I remain, Gentlemen,

Your most obedient Servant,

M. BAILLIE.

*Lower Grosvenor-street;*

*May* 14, 1816.

